# ERN1 and ALPK1 inhibit differentiation of bi-potential tumor-initiating cells in human breast cancer

**DOI:** 10.18632/oncotarget.13086

**Published:** 2016-11-04

**Authors:** Juliane Strietz, Stella S. Stepputtis, Bogdan-Tiberius Preca, Corinne Vannier, Mihee M. Kim, David J. Castro, Qingyan Au, Melanie Boerries, Hauke Busch, Pedro Aza-Blanc, Susanne Heynen-Genel, Peter Bronsert, Bernhard Kuster, Elmar Stickeler, Thomas Brabletz, Robert G. Oshima, Jochen Maurer

**Affiliations:** ^1^ Cancer Research Center, Sanford Burnham Prebys Medical Discovery Institute, La Jolla, CA, USA; ^2^ Department of Visceral Surgery, University Hospital Freiburg, German Cancer Consortium (DKTK), Freiburg, Germany; ^3^ German Cancer Research Center (DKFZ), Heidelberg, Germany; ^4^ Department of Experimental Medicine I, University of Erlangen-Nuernberg, Erlangen, Germany; ^5^ Systems Biology of the Cellular Microenvironment at The DKFZ Partner Site Freiburg, German Cancer Consortium (DKTK), Institute of Molecular Medicine and Cell Research, Albert-Ludwigs-University Freiburg, Freiburg, Germany; ^6^ Technische Universitaet Muenchen, Partner Site of the German Cancer Consortium, Freising, Germany; ^7^ Department of OBGYN, University Clinic Aachen (UKA), Aachen, Germany; ^8^ Department of Surgical Pathology, University Medical Center Freiburg, Freiburg, Germany; ^9^ German Cancer Consortium (DKTK), German Cancer Research Center (DKFZ), Heidelberg, Germany; ^10^ Institute of Pathology, University Medical Center Freiburg, Freiburg, Germany

**Keywords:** ALPK1, ERN1, differentiation therapy, human bi-potential tumor-initiating cells, kinase knockdown

## Abstract

Cancers are heterogeneous by nature. While traditional oncology screens commonly use a single endpoint of cell viability, altering the phenotype of tumor-initiating cells may reveal alternative targets that regulate cellular growth by processes other than apoptosis or cell division. We evaluated the impact of knocking down expression of 420 kinases in bi-lineage triple-negative breast cancer (TNBC) cells that express characteristics of both myoepithelial and luminal cells. Knockdown of ERN1 or ALPK1 induces bi-lineage MDA-MB-468 cells to lose the myoepithelial marker keratin 5 but not the luminal markers keratin 8 and GATA3. In addition, these cells exhibit increased β-casein production. These changes are associated with decreased proliferation and clonogenicity in spheroid cultures and anchorage-independent growth assays. Confirmation of these assays was completed *in vivo*, where ERN1- or ALPK1-deficient TNBC cells are less tumorigenic. Finally, treatment with K252a, a kinase inhibitor active on ERN1, similarly impairs anchorage-independent growth of multiple breast cancer cell lines. This study supports the strategy to identify new molecular targets for types of cancer driven by cells that retain some capacity for normal differentiation to a non-tumorigenic phenotype. ERN1 and ALPK1 are potential targets for therapeutic development.

## INTRODUCTION

Breast cancer represents a heterogeneous disease classified into four intrinsic subtypes luminal A, luminal B, Her2 overexpression and triple-negative [1]. Triple-negative breast cancer (TNBC), not expressing estrogen receptor (ER), progesterone receptor (PR) and human epidermal growth factor receptor 2 (HER2) accounts for 15-20% of all breast cancer cases. In contrast to other subtypes there are no targeted therapies for ER^−^/PR^−^/Her2^−^ TNBC. This very aggressive disease seems to be resistant to a number of conventional chemotherapies and there is recent evidence that this is due to TNBC harboring cancer stem cells (CSCs) [2, 3]. In TNBC a heterogeneous expression of myoepithelial and luminal keratins throughout the tumor is frequently documented [4, 5] giving nourishment to the idea that a co-expressing precursor cell could be a stem cell type in these tumors. Examples of stem cell-like differentiation contributing to the tumor mass include colon carcinoma, teratocarcinoma, ductal carcinoma *in situ*, some brain tumors and certain leukemias [6, 7].

Cell death has generally been used to identify compounds with selective activity on cancer cells [8]. However, strategies to identify compounds and targets involved in cell death generally focus on the most potent, differentially toxic compounds [9]. An alternative to killing these cancer cells was first proposed in the 1980s and was defined as differentiation therapy [10]. In the following years this idea mainly gained recognition in the field of cancers of the hematopoetic system [11, 12] but seemed to be underexplored in the field of epithelial tumors. The idea is to push a mutation-induced proliferative state towards a non-tumorigenic, cytostatic state. The expected lower toxicity of differentiation-inducing agents might permit treatment of earlier stage disease and thereby inhibit progression to invasive cancer.

We have previously identified potent tumorigenic mammary cancer stem cells from a mouse model of basal-like breast cancer that retain the capacity to differentiate to luminal epithelial cells with little or no tumorigenic potential [13]. We hypothesized that the activity of certain kinases may mediate pathways that inhibit differentiation of bi-potential breast tumor-initiating cells towards either a myoepithelial or luminal fate. To test this hypothesis, we used MDA-MB-468, triple-negative breast cancer cells [5], to screen a kinase lentiviral shRNA library. We used keratin immunocytochemistry [14] to identify clones that inhibit proliferation and induce changes in keratin expression consistent with a differentiation-like phenotype. MDA-MB-468 cells, like the previously identified mouse mammary CSCs [13], simultaneously express marker genes of both myoepithelial and luminal mammary epithelial lineages and have been described as bi-lineage type cells [15]. Using immunofluorescent high-content high-throughput screening, we identified 11 kinases that inhibit the differentiation of MDA-MB-468 cells. We present evidence for a role of ERN1 (endoplasmic reticulum to nucleus signaling 1), also known as IRE1 alpha (inositol-requiring 1), and ALPK1 (alpha-kinase 1) in inhibiting the spontaneous differentiation of mammary bi-lineage tumor-initiating cells. The knockdown of either kinase elicits a cytostatic response, shifts marker patterns and phenotype, impairs *in vitro* colony forming ability and dramatically inhibits tumorigenicity. We show that inhibition of ERN1 and ALPK1 restricts anchorage-independent spheroid formation of an additional TNBC cell line and two luminal breast cancer cell lines. Finally, we identify a chemical kinase inhibitor capable of mimicking the effect of knocking down ERN1 in several breast cancer cell lines. This study validates the phenotypic screening strategy and opens the way to re-evaluate kinase inhibitors that may not have been effective in inducing cell death but might still be effective chemotherapeutic agents.

## RESULTS

### Screening human bi-potential tumor-initiating cells for agents inducing differentiation

**Figure 1:**
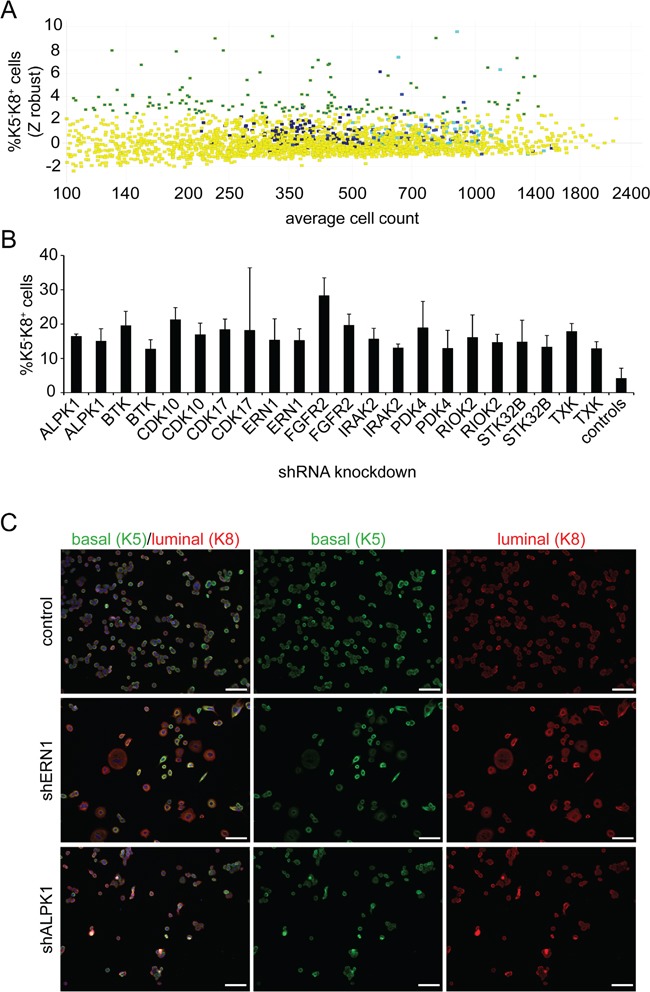
Screening for inducers of differentiation **A.** Summary of the lentiviral screen on MDA-MB-468 cells. Individual dots represent averages of triplicate wells. Yellow indicates treatments that do not reach the threshold. Different shades of blue represent the controls. The green/turquoise color indicates the treatments clearing the threshold of 2.5 standard deviations above the mean of all controls (z-score equals 2.5). X-axis identifies average cell number in triplicate wells. **B.** Representative results of 11 kinases. The averages and standard deviations of triplicate wells are shown. Each bar represents a different vector. **C.** Exemplary images of the lentiviral shRNA treatment of ERN1, ALPK1 and control. MDA-MB-468 cells were analyzed for K5 (green) and K8 (red) expression. Nuclei were visualized by DAPI staining (blue). Scale bars represent 100 μm.

We used bi-potential MDA-MB-468 triple-negative breast cancer cells grown in 2D standard conditions for a high-throughput screening approach to identify kinases that inhibit cancer stem cell differentiation. We targeted 420 kinases using 4-10 lentiviral shRNA constructs per target. This represented 2400 individual, each construct tested in three replicates. Successfully transduced cells were selected for three days utilizing puromycin. After this time, cells were fixed and subjected to myoepithelial keratin 5 (K5) and luminal keratin 8 (K8) immunocytochemistry to quantify cells expressing one or both markers. Representative immunofluorescent images of the positive hits and control cells are depicted in Figure 1C. The increase in potential luminal cells is evident when comparing K5 and K8 immunofluorescent patterns.

To quantitate the imaging results, positive keratin expression scoring thresholds and technical sensitivity were defined by control luminal cell line MCF7 and myoepithelial cell line MCF10A (Supplementary Figure S1) that rarely co-express luminal and myoepithelial keratins. Cells expressing only K5 or K8 (differentiated cells), both (K5^+^/K8^+^, bi-potential tumor-initiating cells) or neither were identified and measured by high-content image analysis. We excluded wells with fewer than 200 cells because low cell numbers are likely due to cytotoxic effects and provide too few events for statistical significance. shRNA constructs were scored as hits if the percentage of K5 or K8 single positive cells was greater than 2.5 standard deviations above the mean of all controls (Figure 1A). The frequency of background positive hits in controls (including empty vector, GFP expressing vector and non-specific control) was 0.0237 (Supplementary Figure S2A). The frequency of two hits for the same gene by chance would be expected to be 0.00056. The frequency of genes with at least two positive results was 0.026 excluding the possibility of chance for those results. Twenty-five of 31 single and double hits that increased K5^−^K8^+^ cells were validated by retesting, but only one of 32 vectors that generated K5^+^ cells was validated. For many of these validated hits, the absolute number of K5^−^K8^+^ cells was increased consistently with induced differentiation, not just selective loss of K5^+^K8^+^ cells. Genes identified by at least two different lentiviral vectors were investigated further.

We identified and validated a total of 11 kinases that induced K5^−^K8^+^ cells by knockdown of at least two differential shRNAs (Figure 1B, Supplementary Table S1). One of the most prominent hits in the MDA-MB-468 screen was FGFR2 (fibroblast growth factor receptor 2). Clones targeting this receptor tyrosine kinase induced a strong differentiation of K5^+^K8^+^ cells towards a K5^−^K8^+^ luminal phenotype. The percentage of K5^−^K8^+^ cells increased from 4% up to 24% on average (Supplementary Figure S3). This supports the validity of the screen as FGFR2 was already known to be involved in breast cancer stem cell maintenance [16]. Two of the top candidates that were further pursued were ERN1 and ALPK1.

### Lentiviral knockdown of ERN1 and ALPK1 reduces mRNA and protein expression

**Figure 2:**
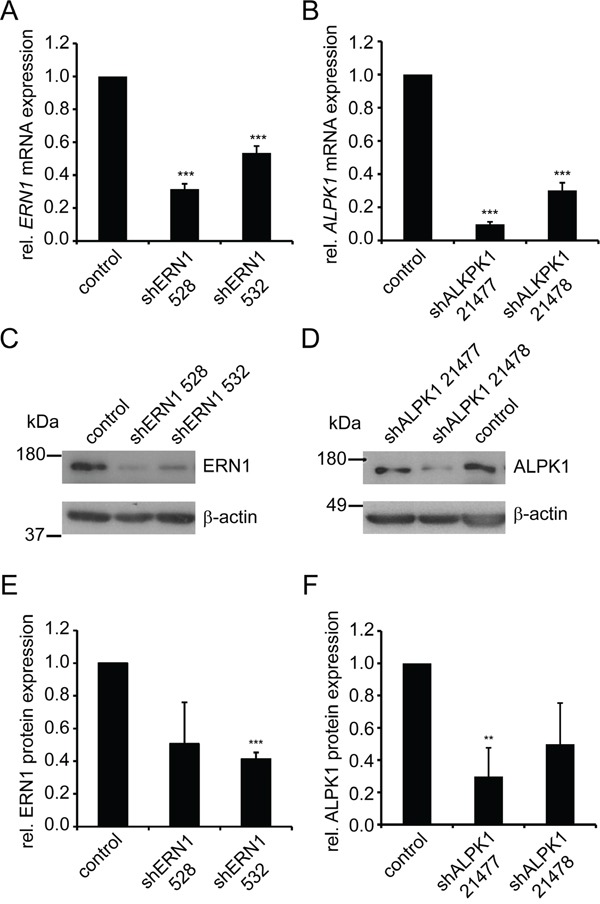
shRNA-mediated knockdown of ERN1 and ALPK1 reduces mRNA and protein expression **A, B.** qPCR data of *ERN1* mRNA (A) and *ALPK1* mRNA (B) expression in MDA-MB-468 cells transduced with lentiviruses carrying vectors with the indicated *shRNAs* or empty vector (control) (n≥6). Expression values were normalized to *HPRT1. ERN1* and *ALPK1* expression in control cells was set to 1. Values are the mean ± SEM. **C, D.** Representative Western blots of ERN1 (C, top) and ALPK1 (D, top) and β-actin (C and D bottom, loading control) of MDA-MB-468 cells transduced with lentivirus carrying vectors with the indicated shRNAs or from control cells transduced with lentivirus carrying an empty vector. **E, F.** Quantification of Western blots. Data are average of at least three independent experiments. ERN1 and ALPK1 expression in control cells was set to 1. Values are the mean ± SEM.

ERN1 and ALPK1 knockdown from either of two shRNAs increased the fraction of K5^−^K8^+^ cells by at least 2.5-fold of the standard deviation of the mean compared to controls (Figure 1B). Depending on the shRNA used, the residual target mRNAs as quantified by qPCR were decreased to 34% and 54% for the two shRNAs targeting ERN1 and 9% and 36% for the ALPK1 shRNAs compared to the control (Figure 2A and 2B). Western blotting confirmed the knockdown of ERN1 and ALPK1 on protein level (Figure 2C-2F). Since efficiency of the knockdown varied between several virus preparations the knockdown at the mRNA level was confirmed by qPCR for every experiment (Supplementary Figure S4).

### Knockdown of ERN1 and ALPK1 affects cell morphology, decreases myoepithelial markers and stabilizes a luminal phenotype

**Figure 3:**
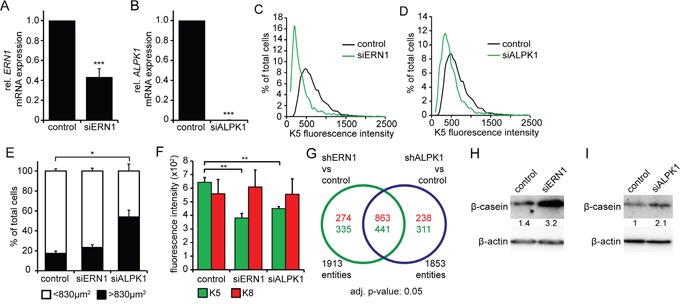
siRNA knockdown of ERN1 and ALPK1 enriches for a luminal cell type **A, B.** qPCR data of *ERN1* mRNA (A) and *ALPK1* mRNA (B) expression in MDA-MB-468 cells transfected with siRNA targeting either *ERN1* or *ALPK1* mRNA or *GFP* (control) (n=3). Expression values were normalized to *HPRT1. ERN1* and *ALPK1* expression in control cells was set to 1. Values are the mean ± SEM. **C**, **D.** K5 fluorescence intensity of single MDA-MB-468 cells after ERN1 (C) and ALPK1 (D) knockdown compared to control cells. Depicted are representative curves. **E.** Percentage of MDA-MB-468 cells smaller or bigger than 830 μm^2^ (n=3). Values are the mean ± SEM. **F.** K5 and K8 expression in big (>830 μm^2^) MDA-MB-468 cells after ERN1 or ALPK1 knockdown compared to controls (n=3). Values are the mean ± SEM. Statistical significances in this figure were evaluated by two-tailed Student's *t*-test. **G.** Venn diagram of up- and down-regulated genes from shRNA-mediated knockdowns of ERN1 and ALPK1. **H, I.** Representative Western blots detecting β-casein in MDA-MB-468 cells transfected with siRNA targeting ERN1 and ALPK1, respectively. β-actin was used as loading control.

To confirm the effects of the shRNA-mediated ERN1 and ALPK1 knockdown, we also used ERN1- and ALPK1-specific siRNAs. Using siRNA instead of the viral constructs showed a better knockdown efficiency for ALPK1, but not ERN1 (Figure 3A and 3B). The induction of differentiation was confirmed by automated immunocytochemistry which showed a clear decrease of K5^+^ cells consistent with a K5^−^K8^+^ luminal cell profile (Figure 3C and 3D, Supplementary Figure S5A, S5B and S5D). Sample images shown here also identify a phenotypic change in cell culture: an increase in cell size accompanying a stretched epithelial morphology (arrowheads, Supplementary Figure S5E). This phenotype remained stable during cell culture sub-cultivation. The change in cellular morphology was more pronounced in the ALPK1 knockdown cells (Supplementary Figure S5E, siALPK1) where we measured a significant increase in cell size. The ratio of small cells (<830 μm^2^) to big cells (>830 μm^2^) shifted significantly in ALPK1 knockdown cells (Figure 3E). The identification of the large cells as differentiated cells was reinforced by the significant loss of K5 fluorescence in large but not small cells (Figure 3F, Supplementary Figure S5C).

To assess the effect of siRNA-mediated ERN1 and ALPK1 knockdown on cell death, we performed AnnexinV-PI FACS analysis after 3 days. Analysis of control and knockdown MDA-MB-468 cells showed no difference in number of necrotic or early apoptotic cells. While late apoptosis was not altered in ALPK1 knockdown cells, number of late apoptotic ERN1 knockdown cells was slightly decreased compared to control cells (Supplementary Figure S5F). The change in keratin pattern and morphology in viable knockdown cells is consistent with a differentiation-like process not selective cellular death.

### ERN1 and ALPK1 knockdown leads to similar luminal-like differentiation

Two individual transcriptome analyses were performed to examine the alteration of gene expression patterns in transient (siRNA) and stable (shRNA) MDA-MB-468 knockdown cells and to compare the similar luminal-like differentiation observed by immunocytochemistry (Figures 1C and Supplementary Figure S5E). We compared two biological independent replicates of shERN1 and shALPK1 in addition to two biological independent replicates of siERN1 and siALPK1 using Illumina HT-12 Expression Bead Chips.

We determined the differentially regulated genes from ERN1 and ALPK1 knockdown compared to shRNA control (log2 fold change cutoff >0.5, adjusted p-value <0.05). Relative to the shRNA control we found 1137 (776) genes to be significantly up- (down-) regulated in shERN1 and 1101 (752) up- (down-) regulated in shALPK1 (Figure 3G). Moreover, both knockdowns shared 863 up- and 441 down-regulated genes.

A gene set enrichment analysis (GSEA) (PMID: 19473525) identified that processes involved in proliferation and cell division were down-regulated upon siALPK1 treatment which might be expected from a process inducing differentiation (Supplementary Figure S6). The analysis also confirmed the luminal shift in key regulated genes (Supplementary Figure S7).

Although the gene expression profile does not match fully differentiated luminal or alveolar mammary epithelial cells, we analyzed expression of β-casein, a milk protein preferentially expressed by differentiated alveolar cells. We found increased expression of β-casein in both knockdown cells (Figure 3H and 3I) with a stronger upregulation in cells transfected with siERN1 (Figure 3H). Taken together, the transcriptome response of cells after ERN1/ALPK1 knockdown are consistent with the phenotypic changes towards a more differentiated cell type with alterations in both cytoskeletal (keratins) and luminal cell protein content (β-casein). In addition, β-casein expression indicates a cell fate shift towards an alveolar mammary epithelial cell type.

It was interesting to note that *XBP1* mRNA was down-regulated by both ERN1 and ALPK1 knockdown using shRNA. Since *XBP1* is a known target of ERN1 [17] and implicated in triple-negative breast cancer [18], we tested regulation of the gene by qPCR. We found total *XBP1*, unspliced as well as the alternatively spliced variant, down-regulated in ERN1 or ALPK1 MDA-MB-468 knockdown cells compared to control cells (Supplementary Figure S8).

### Knockdown of kinases leads to reduced colony and tumor formation

**Figure 4:**
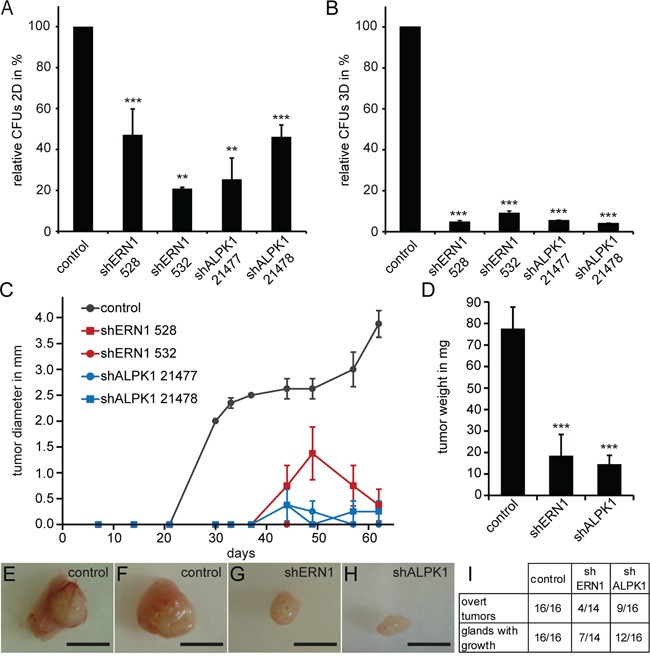
MDA-MB-468 cells with an ERN1 or ALPK1 knockdown form fewer colonies in 2D and 3D and develop fewer and slower growing tumors **A.** Colony forming capacity depicted as relative colony forming units (CFUs) of control, shERN1 or shALPK1 transfected MDA-MB-468 cells in a 2D assay (n=3). Depicted are the mean values ± SEM. Statistical significances were evaluated by Tukey's test. **B.** CFUs of control, shERN1 or shALPK1 transfected MDA-MB-468 cells in a 3D assay, embedded in Matrigel (n=3). Depicted are the mean values ± SEM. Statistical significances were evaluated by Tukey's test. **C.** Growth curves for tumor transplants from 5×10^5^ ERN1 knockdown, ALPK1 knockdown or control MDA-MB-468 cells (n=4, per treatment). Depicted are the mean values ± SEM. **D.** Average tumor weight in milligrams from ERN1 knockdown, ALPK1 knockdown or control cell transplants (n≥14). Depicted are the mean values ± SEM. Statistical significances were evaluated by two-tailed Student's *t*-test. **E, F.** Exemplary control tumors. **G, H.** Exemplary ERN1 (G) and ALPK1 (H) knockdown tumors. Scale bars represent 5 mm. I. Analysis of cell transplants from 5×10^5^ MDA-MB-468 cells. Overt tumors describe growth visible to the naked eye in the mammary gland of the animal. Glands with growth describe cellular growths that could only be identified by carmine alum staining of the glass-mounted mammary gland as seen in Supplementary Figure S9.

We analyzed the effect of ERN1 and ALPK1 knockdown in MDA-MB-468 cells on proliferation and colony forming ability *in vitro* and *in vivo*. For these long-term experiments, lentiviral shRNA knockdown was used. We hypothesized that a more differentiated luminal cell would be unable to form colonies from single cells. ERN1 or ALPK1 knockdown cells formed significantly fewer colonies in 2D (Figure 4A) and 3D (Figure 4B). The 3D colony formation assay which is widely used to assess cancer stem cell self-renewal *in vitro* was more sensitive to the suppression of ERN1 and ALPK1 than the 2D assay. Furthermore, the colony forming efficiency was dependent on the knockdown efficiency of either gene (Supplementary Figure S9).

The current gold standard of testing tumorigenicity is the orthotopic transplantation of the cells into immunocompromised mice. We therefore performed orthotopic transplants of 5×10^5^ MDA-MB-468 cells into the mammary glands of NOD/SCID mice and monitored the tumor growth over a period of two months. The control tumors started to develop between day 21 and day 30 and grew consistently over the whole period of the experiment (Figure 4C). Onset of tumor growth from knockdown cells was delayed until day 44, at which time small growths were detectable by caliper in some of the cohort. Transplants of ERN1 knockdown cells kept growing for additional 5 days before starting to regress and reduce in volume again. Mice were sacrificed after 62 days due to exceeding the maximum allowed tumor burden in the control animals. Tumors from control cells were approximately 4-5 times larger than tumors from knockdown cells (Figure 4C and 4E-4H). This was also confirmed by the average tumor weight at the end of the experiment. While control tumors averaged at about 80 mg, shERN1 cell-derived tumors had an average weight of 20 mg and shALPK1 derived tumors about 17 mg (Figure 4D). Only 4 out of 14 transplants from shERN1 cells and 9 out of 16 transplants from shALPK1 cells developed into a palpable tumor compared to 100% palpable growth from control cells (Figure 4I). Mammary glands without palpable tumor growth were mounted and growths were identified by carmine alum staining (Supplementary Figure S10).

### Tumors derived from knockdown cells show signs of differentiation early but not later in tumor development

**Figure 5:**
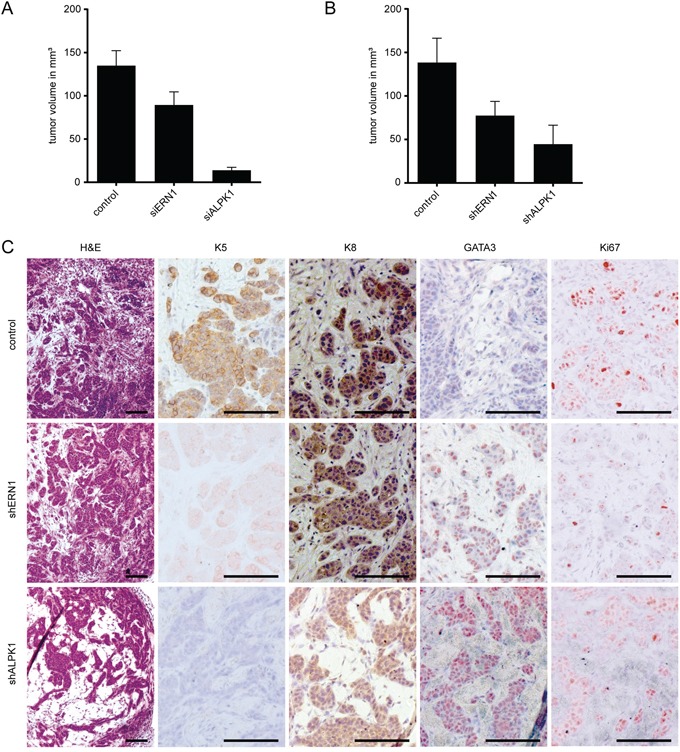
Early ERN1 and ALPK1 knockdown tumors differ in expression pattern or phenotype from control tumors **A.** Volumes of tumors (in mm^3^) derived from control, siERN1 or siALPK1 transfected MDA-MB-468 cells 16 days after transplantation. Depicted are the mean values ± SEM. **B.** Volumes of tumors (in mm^3^) derived from control, shERN1 or shALPK1 transfected MDA-MB-468 cells 16 days after transplantation. Depicted are the mean values ± SEM. **C.** The panel depicts representative images of tumors sections derived from control, shERN1 and shALPK1 transfected MDA-MB-468 cells (from top to bottom) 16 days after transplantation. Histochemical staining is indicated on the top from left to right: H&E (hematoxylin and eosin), K5 (keratin 5), K8 (keratin 8), GATA3 and Ki67. Scale bars represent 100 μm.

In order to determine the growth pattern of the knockdown cells during the early phase of tumor formation we isolated four individual transplants of 4x10^6^ MDA-MB-468 cells either transfected with siERN1, siALPK1 or shERN1, shALPK1 and appropriate controls 16 days after transplantation. In this early phase of tumor development, the effect on the knockdown cells in culture could be reproduced *in vivo*. The developing control tumors measured on average 135 mm^3^ in volume for siRNA (Figure 5A) and 138 mm^3^ for shRNA (Figure 5B). The knockdown growths developed slower and were smaller on average. Tumor growths derived from siERN1 knockdown cells measured on average 89 mm^3^ and from siALPK1 knockdown cells on average 14 mm^3^ (Figure 5A). Similarly, tumor growths derived from shERN1 knockdown cells measured on average 77 mm^3^ and from shALPK1 knockdown cells on average 45 mm^3^ (Figure 5B).

Immunohistochemical analysis of the tumors developing from shRNA knockdown cells showed decreased K5 and Ki67 expression and increased GATA3 expression (Figure 5C), replicating the phenotype of cells documented *in vitro*. Keratin 8 expression remained unchanged in all tumors. These results are consistent with down-regulation of K5 expression and maintenance of K8 expression.

The delayed growth of shERN1 and shALPK1 tumors may be due to loss of effective suppression of the targets during growth *in vivo* without continued puromycin selection. We measured expression of the targeted genes by qPCR and compared knockdown and control cells before the transplantation and in late developing tumors. We found a strong re-expression of ERN1 and ALPK1 in the tumor cells arising from an ERN1 knockdown after late tumor passage (Supplementary Figure S11B). Therefore, growth *in vivo* appears to select strongly for the re-expression of the targeted genes and subsequent tumor growth. Histological analyses of late tumors resulting from transplantation from either knockdown or control cells could not be distinguished (Supplementary Figure S11A). All late tumors were determined to be grade 3 (poorly differentiated) and showed no differences in immunohistochemistry for K5, K8 or GATA3 (Supplementary Figure S11A). A strong selection against the continued suppression of ERN1 or ALKP1 occurs during tumor development *in vivo* over time.

### The differentiation response upon ERN1/ALPK1 knockdown is breast cancer specific

**Figure 6:**
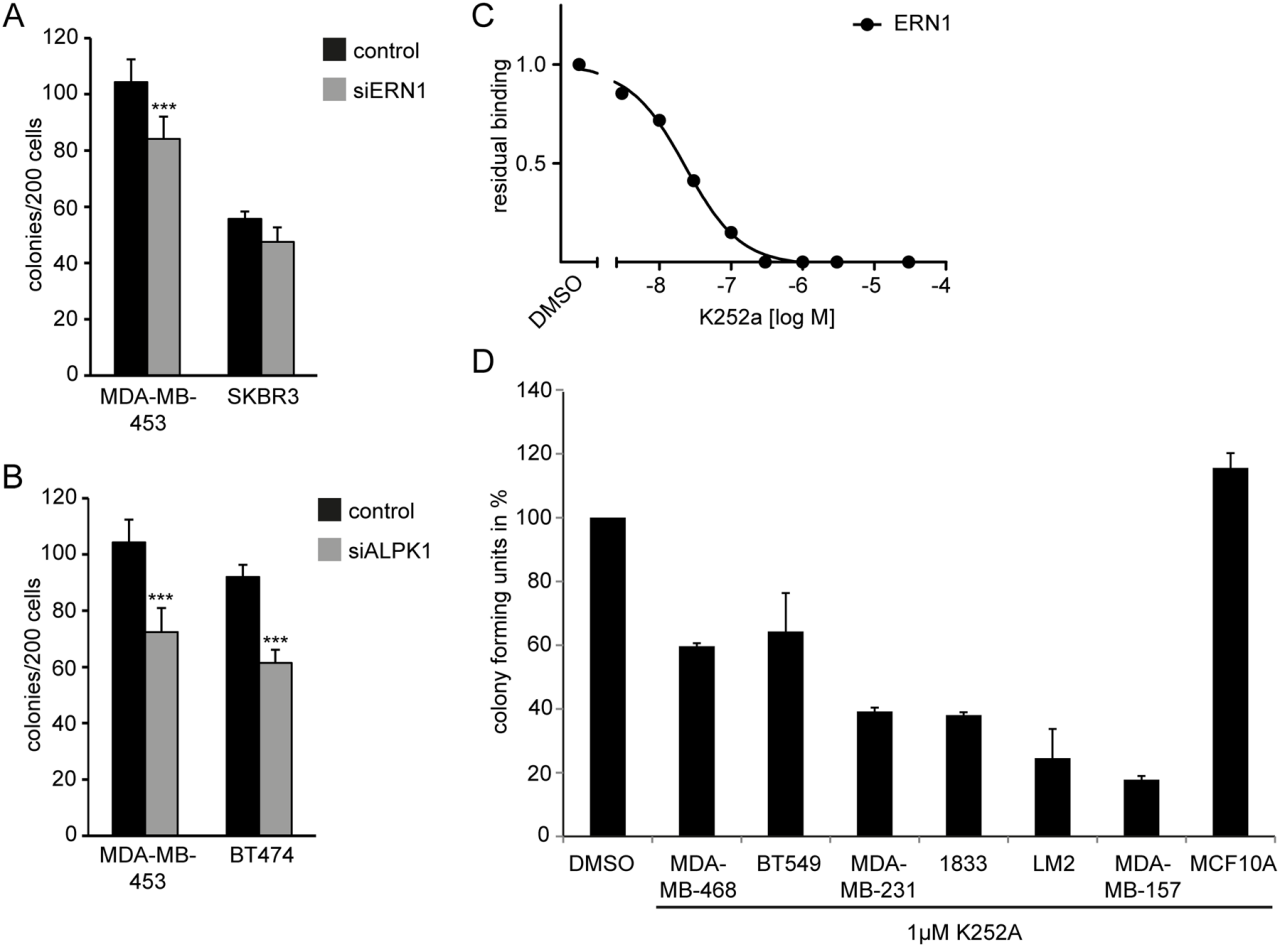
ERN1 or ALPK1 knockdown affects colony forming and proliferative capacity of mammary cancer cell lines **A, B.** Colony forming capacity depicted as absolute colony forming units (CFUs) per 200 cells of control, siERN1 or siALPK1 transfected TNBC cells in a 2D assay (n=3). Values are the mean ± SEM. **C.** Binding activity assay for the K252a inhibitor to the ERN1 Kinase. **D.** Anchorage-independent growth in TNBC cell lines treated with DMSO alone or 1 μM K252a inhibitor over 3 days. Colony forming capacity is depicted in % versus DMSO treatment set to 100%. Data were collected from three individual experiments with eight individual measurements per experiment (n=24). Error bars represent SEM (1833=MDA-BoM1833, LM2=MDA-MB231 LM2).

The restriction of tumorigenicity and 3D colony formation of MDA-MB-468 cells by ERN1 and ALPK1 knockdown might be due to cell type-specific developmental programs or might be a less specific consequence of slowing down the growth of the cells. We challenged multiple cancer cell lines with ERN1 or ALPK1 siRNA and assessed proliferation and colony forming ability from single cells. Breast cancer cell lines MDA-MB-453 (triple-negative) and SKBR3 (luminal) exhibited a reduced colony formation when transfected with siRNA targeting ERN1 (Figure 6A). Transfection with siRNA against ALPK1 reduced colony forming ability of MDA-MB-453 (triple-negative) and BT474 (luminal) (Figure 6B).

There are no reported kinase inhibitors selective for ERN1 or ALPK1. However, one broadly selective tyrosine kinase inhibitor that is also active against ERN1 is K252a. The binding constant for ERN1 was about 40 nM (Figure 6C). The K252a inhibitor is a staurosporine derivative targeting ERN1, CamK, PKA, and TRKs among other kinases. We identified a dose of 1 μM as tolerated by normal mammary cells (MCF10A, Figure 6D) and evaluated 3D colony forming ability of different cancer cell lines under treatment with K252a. The ability of MDA-MB-468 cells to form colonies in anchorage-independent growth assays was reduce by half (Figure 6D). Furthermore, other TNBC cell lines were equally or stronger affected by inhibitor treatment (Figure 6D) reducing the colony forming capacity down to 20% compared to DMSO-treated control cells.

Finally, K252a was examined for its ability to induce β-casein expression in MDA-MB-468 cells. After 72 hours, treatment with 1 μM K252a doubled β-casein protein amount compared to control cells (Supplementary Figure S12).

## DISCUSSION

We used a high-content high-throughput imaging method to identify kinases that support the stem cell character of bi-potential tumor-initiating cells from triple-negative breast cancer. This assay is based on the co-expression of K5 and K8 by these cells and the loss of K5 during progression to a luminal-like cell fate. A previous chemical screen of breast cancer stem cells was based on growth inhibition or cell death relative to normal mammary epithelial cells [9]. Our phenotypic screen identified candidates that may be key contributors to tumorigenicity even though their suppression did not induce cell death. This approach validates phenotypic screening in contrast to conventional assays targeting cell viability.

We showed here that restricting the expression of ERN1 and ALPK1 independently leads to a loss of anchorage-independent growth and reduced tumorigenicity. This is associated with an increased cell size and altered morphology, expression of luminal epithelial marker genes and decreased expression of myoepithelial marker genes. However, mRNA expression analysis of ERN1/ALPK1 knockdown cells did not closely match expression profiles of mature mammary ductal cells. This incomplete differentiation may reflect the strong selective pressure exerted on this cell line for growth during the evolution of the original tumor and many years in monolayer culture. Support for this idea is also evident from the strong selective pressure in the xenograft experiment. Even when the vast majority of cells seem to have entered a state of beginning differentiation, some cells are able to counter the knockdown effect and are sufficient to revive tumor growth long-term. A sustained and potent inhibition of ERN1 or ALPK1 may be necessary to be most effective. Mouse CSCs from a much more differentiated tumor are capable of greater differentiation in culture and *in vivo* [13]. In addition, Bosutinib, a tyrosine kinase inhibitor, induced differentiation of MMTV-PyMT tumors resulting in both epidermal differentiation and greater luminal cell maturation [19]. While MDA-MB-468 cells may be too abnormal to adopt a mature luminal state, it is encouraging to discover that loss of tumorigenicity appears to be a relatively early step in the pathway towards ductal differentiation. The relevance of ERN1 and ALPK1 as potential therapeutic targets was reinforced by an effect of the respective siRNAs on colony formation of other breast cancer cell lines.

The induced phenotypic change of MDA-MB-468 cells is consistent with the discovery of a luminal progenitor as the cell of origin for triple-negative breast cancer [20]. As seen in our data, MDA-MB-468 cells could only be directed towards luminal differentiation, consistent with a partially committed, differentiated fate. This limited developmental fate is still consistent with the definition of a cancer stem cell that is capable of generating tumor heterogeneity. However, this breast cancer stem cell might be considered more of a bi-potential progenitor than a stem cell in a developmental sense [21]. Normal mammary stem cells, capable of generating luminal ducts, alveolar end buds and myoepithelial cells, have greater developmental potential.

ALPK1 is part of the membrane transport machinery in the lipid-raft dependent pathway; located in the Golgi-vesicles it has an important function in spatial organization of proteins in epithelial cells [22–25]. A very recent study shows evidence of a correlation of ALPK1 mRNA expression with tumorigenesis in lung and colorectal cancer [29]. The authors identify several novel mutations in ALPK1 in a specific Taiwanese cohort of patients and propose these SNPs to have prognostic value for susceptibility and prognosis [29].

ERN1 or IRE1 has a well-documented role in the protein degradation pathway, almost uniquely executed through alternative splicing of Xbp1. Its role in cancer progression was also documented along this axis, showing an activation of the pathway inducing proliferation in colorectal carcinoma cells [26]. It was identified as a druggable target and potential therapeutic option in multiple myeloma [27, 28] and seems to be amplified in 8% of invasive breast cancers [18, 19]. Nevertheless most of these studies focused on the role of Xbp1 alternative splicing. Our data indicate a potential Xbp1 effect that is splicing-independent.

It will be interesting to determine how ERN1 and ALPK1 mediate the same or separate pathways that inhibit differentiation and promote tumorigenicity. Gene expression patterns of shERN1 and shALPK1 cells overlap remarkably, suggesting roles within the same pathway. Interestingly, levels of both *XBP1* mRNA isoforms (*XBP1u* and *XBP1s*) are lower in cells transfected with shERN1 or shALPK1. It has been recently shown that *XBP1* alternative splicing was higher in primary TNBC patient samples compared to non-TNBC samples and that interfering with XBP1 expression results in lower tumorigenicity and colony formation of TNBC cell lines [18]. The knockdown of ERN1 decreased the amount of *XBP1s* (alternatively spliced mRNA) as expected and decreased the amount of *XBP1* total RNA as well. It is currently unclear how the repression of ALPK1 regulates *XBP1* on the RNA level. Further studies are needed to gain mechanistic insight into the ERN1/ALPK1/XBP1 hypothesis.

We chose to target kinases in this phenotypic screen of TNBC cells because kinases are good candidates for the development of selective chemical inhibitors. Currently, there are no selective kinase inhibitors for ERN1 or ALPK1 available and it is not yet clear whether the kinase activity of the candidate target proteins is essential. However, a preliminary screen of a library of kinase inhibitors identified chemical candidates which increased the population of MDA-MB-468 K5^−^K8^+^ cells (Supplementary Figure S2C). In addition, we could show that the K252a inhibitor decreases anchorage-independent growth in MDA-MB-468 and several other TNBC cell lines. As most kinase inhibitors are commonly active on multiple kinases, future experiments will be necessary to determine if any of the kinases identified in the differentiation screen are inhibited by these chemicals. Combinatorial screens of clinically approved inhibitors, targeting for example FGFR2 like Dovitinib (Ariad) or Ponatinib (Novartis), could benefit patients in the future. The patient-derived bi-potential tumor-initiating cell cultivation combined with screening of pharmaceutical libraries to identify the most effective compounds for a particular tumor could be part of future cancer treatments in the clinic.

Differentiation therapy could provide several benefits for patients suffering from CSC-driven cancers. On the one hand, it would provide a different kind of cancer treatment likely with less severe side effects than chemotherapy or radiation. On the other hand, recent studies addressing tumor growth patterns identify a need to search for alternatives to therapies resulting in tumor cell death. Simulations based on actual tumor growth patterns showed how detrimental the influence of apoptotic cell death in tumor growth patterns can be when considering the so-called self-seeding metastases [29–31]. Freeing up space in a solid tumor by cell death allows for faster and more aggressive growth [32]. In contrast, differentiation or a differentiation-like process stabilizes a cellular phenotype and restricts cellular space for cancer cells to expand into, keeping the cellular composition of the tissue intact. This could prove beneficial in a therapeutic approach combining minimal invasive surgery and differentiation therapy.

Tumor heterogeneity, including the CSC paradigm, demands a more individualized therapy including in-depth knowledge about the tumor in question. We propose differentiation therapy may be particularly appropriate for tumors driven by CSCs and for arresting the progression of early stage tumors. Further studies are needed to determine how advanced a cancer can be and still respond to a stimulus to differentiate.

## MATERIALS AND METHODS

### shRNA screen

The shRNA screen was performed using a subset of SIGMA's Lentiviral Human Kinome Mission shRNA library targeting 481 kinases with an average of 4.8 shRNA constructs per target. For viral production, 50 ng of each construct were combined with PCMV dR8.74 (35 ng) and PMD.G-VSVG (17.5 ng) packaging plasmids and transfected into 293FT cells using X-tremegene HP (Roche) in 96-well format in collagen-coated plates. Twenty-four hrs after transfection, culture media were replaced and viral supernatants were collected 48 hrs later. MDA-MB-468 cells were purchased from ATCC and cultivated in standard medium (DMEM, 10%FBS, 1%Pen-Strep). For screening, MDA-MB-468 cells were plated on gelatin-coated (0.1%) black, clear bottom 384-well plates (Greiner Cat.# 781092) at 1200 cells/well in standard medium. Twenty-four hrs later, cells were transduced with 20 μl of viral supernatants in the presence of polybrene (estimated average titer of 1x10^6^ infective particles/ml). After 24 hrs, cells were washed (405 LS Microplate Washer, Biotek) with media to remove virus and standard medium containing puromycin (1 μg/ml) was added (Microflo Select Dispenser, Biotek). Cells were selected for 72 hrs. At the end of selection, plates were washed with PBS once and fixed in ice-cold methanol for 10 min at −20°C. Cells were stored in methanol at −20°C until immunofluorescence labeling was performed.

Every screened plate contained several dilutions of control viral particles (non-specific neo-resistant, non-specific puromycin-resistant, Turbo-GFP (infection control) and empty lentiviral vector), totaling 56 controls per plate (1176 controls in total for the screen). In addition each plate contained MCF7 and MCF10A control cells for immunofluorescence gating purposes (168 in total) (Supplementary Figure S1).

Plates were imaged on the Opera QEHS (PerkinElmer, Inc.) confocal high-content imaging system at 10x (0.4 NA) with 8 images per well. Alexa Fluor^®^ 488, Alexa Fluor^®^ 568 and DAPI were acquired using 488 nm, 561 nm, and 405 nm laser excitations; 540/75, 600/40, and 450/50 emission filters; and 40 ms, 600 ms and 120 ms exposure times, respectively. Acquired images were transferred to the Columbus™ Image Data Storage and Analysis System (PerkinElmer, Inc.) and subsequently analyzed with a custom Acapella (PerkinElmer, Inc.) analysis script. Briefly, nuclear detection (Acapella Nuclear Detection Library, PerkinElmer, Inc.) and cytoplasm detection (Acapella Cytoplasm Detection Library, PerkinElmer, Inc.) was performed. Cells with pyknotic nuclei were removed from further analysis, followed by measurement of intensity of Alexa Fluor^®^ 488 and Alexa Fluor^®^ 568 in the cell body. 2.5 standard deviations above the mean intensity of MCF7 (Alexa Fluor^®^ 488) and MCF10A (Alexa Fluor^®^ 568) control wells, across the entire plate set, were used as a threshold. Counts and percentages of K5^+^, K8^+^, K5^+^/K8^+^, K5^−^/K8^−^, K5^+^/K8^−^, K5^−^/K8^+^ populations were calculated per well.

### Kinase inhibitor library screen

MDA-MB-468 cells were plated on gelatin-coated (0.1%) black, clear bottom 384-well plates (Greiner Cat.# 781092) at 1200 cells/well in standard medium. 24 hrs later cells were treated with 1 μM of the EMD Inhibitor Select^TM^ Kinase Library (244 compounds). Cells were incubated at 37°C for 72 hrs. After selection, plates were washed with PBS once and fixed in ice-cold methanol for 10 min at −20°C. Cells were stored in methanol at −20°C until immunofluorescence labeling was performed. Labeling and detection were performed as described above.

### Lentivirus preparation and knockdown

Lentivirus was packaged by co-transfection of constructs with the 3rd generation packaging plasmids pCMVdR8.74 and pMDVSVG with X-treme gene HP (Roche, # 06366236001) into 293FT cells. Medium was replaced with Ultraculture (Biowhittaker, #BE12-725F) after 24 hrs. 2 ml of virus preparation per well were harvested 48 hrs after transfection. Suspensions were centrifuged at 500 g for 10 min at room temperature and the supernatant was filtered through a 0.45 μm syringe filter. Virus was used after 24 hrs when stored at 4°C; otherwise aliquots were stored at −80°C for long term storage.

The virus titer was determined via qPCR (abm qPCR Lentivirus Titration Kit, #LV900) and the cells seeded 24 hrs prior to transfection in suitable cell numbers to achieve a multiplicity of infection (MOI) of approximately 100. After a 3 day selection period with medium containing 1 μg/ml puromycin, the cells were used for further analyses.

### Transplants

All mouse handling and experiments were performed in accordance of German Animal Welfare regulations and approved by the local authorities. NOD/SCID females (4-5 week old) were anesthetized using an isoflurane inhalator. A small sagittal incision (no longer than 1.5 cm) on the shaved and sterilized abdomen allowed access to the mammary glands #4 on both sides. Tumor cells were mixed with 1 million irradiated fibroblasts (human foreskin fibroblasts HF27, p11) each and suspended in a 1:1 mixture of Matrigel and DMEM/10%FBS. The volume of each transplant was 20 μl per gland, containing 5×10^5^ or 4×10^6^ tumor cells (knockdown or control) and 1 million fibroblasts.

The transplant was injected into the mammary fat pad of the #4 gland on both sides of the animal using a 1 ml syringe with a fine needle. Each transplant was localized distal of the lymph node in the gland. Surgical incisions were sealed by stitching with a 5/0 thread (Ethicon, Z995). Animals were monitored weekly for animal weight and tumor growth, which was determined by caliper.

### Paraffin embedding and immunohistochemistry

Specimens were fixed in 4% PFA/PBS overnight at 4°C. Samples were dehydrated in an ethanol series (30%, 50%, 70% and 100%) for 2 hrs each, followed by two 10 min incubations in 100% Rotihistol, and were then transferred to paraffin overnight. Samples cast into paraffin blocks were sectioned at 5 μm using a RM2255 microtome (Leica Biosystems, Wetzlar). Samples were deparaffinized in Rotihistol and rehydrated in a descending ethanol series.

For H&E staining slides were incubated for 5 min in Mayer's hemalum solution (1:10, Merck, Darmstadt) and washed in distilled water. The staining was further enhanced by incubation in warm tap water. Subsequently, slides were stained for 5 min in 0.15% eosin and after dehydration mounted using Roti-Histokit (Roth, Karlsruhe).

For immunohistochemistry, epitope retrieval was performed by boiling samples for 20 min in 10 mM citrate buffer with 0.05% Tween20 (pH 6.0). To block endogenous peroxidases, slides were treated with 3% H_2_O_2_ (DAKO, #S2032) for 30 min. After washing for 15 min in TBST (0.05 M Tris-HCl, 0.9% Sodium chloride, 0.05% Tween20, pH 7.6), slides were incubated with primary antibody in DAKO antibody diluent (DAKO, #S7653) overnight at 4°C. The following primary antibodies were used: rabbit anti-K5 (1:200, Covance, PRB-160P), mouse anti-K8 (1:200, Sigma, #C5301), rabbit anti-GATA3 (1:100, Sigma, #HPA029731), rabbit anti-Ki67 (1:100, abcam, #ab16667). After washing for 15 min in TBST, slides were incubated with EnVision+System-HPR polymer anti-mouse (DAKO, #K4001) or anti-rabbit (DAKO, #K4003) for 1 hr at RT. Slides were washed in TBST and proteins visualized by staining with 3-amino-9-ethylcarbazol (AEC) solution. Finally, slides were counterstained with Mayer's hemalum solution (1:5) for 1 min and mounted with Kaiser's glycerol gelatin (Merck, Darmstadt).

Subsequently, protein expression was documented using the Olympus BX61 microscope.

### Carmine alum staining

Mammary glands were excised carefully, mounted onto standard microscope glass slides and fixed in Carnoy's fixative (6 parts 100% ethanol, 3 parts CHCl_3_, 1 part glacial acid) overnight at room temperature. Slides were washed in 70% ethanol for 15 minutes. Ethanol was changed to distilled water gradually, with a final 5 minute rinse in water. Slides were incubated in carmine alum solution (1g carmine (Sigma C1022) and 2.5g aluminum potassium sulfate (Sigma 7167) dissolved in 500ml distilled water, boiled for 20 minutes) overnight. Slides were cleared in 70%, 95%, 100% ethanol and Xylene consecutively and incubated in Xylene overnight. Next day, slides were mounted in Permount^©^.

### Transient knockdown of ERN1 and ALPK1 using siRNA

Cells were transfected in suspension with control nonspecific or GFP-targeting siRNAs or siRNAs targeted against *ERN1* or *ALPK1* (10 nM) using Lipofectamine^®^ RNAiMAX Transfection Reagent (Life Technologies). The following siRNAs were used: siGFP (M. Truss, Berlin), Silencer^®^ Negative Control #1 (Ambion, Life Technologies, #AM4611), ERN1 pre-designed Silencer^®^ siRNA (Ambion, Life Technologies, #289308), ALPK1 validated Silencer^®^ siRNA (Ambion, Life Technologies, #1074). After 24 hrs, transfection medium was replaced by normal growth medium and the cells were cultured for further 48 hrs. Subsequently, cells were either lysed for RNA extraction, seeded for colony formation assays or fixed in ice-cold methanol for immunocytochemistry.

### Immunofluorescence labeling and *scan^R* microscopy

For immunofluorescence labeling, cells were fixed in ice-cold methanol at −20°C overnight, washed in PBS and permeabilized with TBST (20 mM Tris, 150 mM NaCl, 0.05% Tween-20). After blocking in 1 mg/ml ovalbumin in PBS for 1 hr at room temperature, the cells were incubated with primary antibody diluted in blocking solution for 1 hr at 37°C. The following primary antibodies were used: rabbit anti-K5 (1:250, Covance, PRB-160P), mouse anti-K8 (1:250, Sigma, #C5301). After washing in PBS, the cells were treated with secondary antibody diluted in blocking solution for 30 min at 37°C. The following secondary antibodies were used: Alexa Fluor® 488 Donkey Anti-Rabbit (1:500, Molecular Probes®, Life Technologies), Alexa Fluor^®^ 568 Donkey Anti-Mouse (1:500, Molecular Probes^®^, Life Technologies). Counterstaining of nuclei was performed by storage in DAPI buffer (10 mM Tris-HCl, pH 7.4, 10 mM EDTA, 0.1 M NaCl) containing 0.1 μg/ml DAPI (Sigma).

Subsequently, cells were screened using the Olympus scan^R microscope. Fluorescence intensities and cell sizes were further analyzed using the scan^R analysis software.

### RNA isolation

Total RNA was isolated from sub-confluent cultures grown in 6-well plates using the RNeasy Plus Kit from Qiagen, applying the Qiagen Supplementary Protocol: Purification of miRNA from animal cells using the RNeasy^®^ Plus Mini kit and RNeasy MinElute^®^ Cleanup Kit (Protocol 1). RNA concentration was quantified using the NanoDrop 2000 (Thermo Scientific).

### cDNA synthesis and qPCR

Reverse transcription was performed using the RevertAid First Strand cDNA Synthesis Kit (Thermo Scientific) according to the manufacturer's manual. A maximum of 2 μg total RNA was reverse transcribed.

qPCR was executed using gene-specific primers, UPL probes (Universal Probe Library, Roche) and TaqMan^®^ Universal Master Mix II, no UNG (Applied Biosystems, Carlsbad, CA) on a Roche Light Cycler 480 applying the following cycling parameters: 95°C 10 min, 40 cycles of 95°C 15 s, 60°C 1 min. Relative RNA expression to the corresponding control was calculated with the Pfaffl quantification method [33] and *HPRT1* was used as normalization control. The following primers and UPL probes were used:

### Microarray

In the first array, we analyzed RNA with an Illumina HumanHT-12 v4 Expression BeadChip Kit using the manufacturer's BeadArray Reader and collected primary data using the supplied scanner software. Data analysis was done in three stages.

**Table:** 

	forward 5′-3′	reverse 5′-3′	UPL
*ALPK1*	tgaccaccatttgctgtcc	acgtgccacggatattcac	#08
*ERN1*	gaagcatgtgctcaaacacc	tctgtcgctcacgtcctg	#50
*HPRT1*	tgaccttgatttattttgcatacc	cgagcaagacgttcagtcct	#73
*XBP1_total*	ggagttaagacagcgcttgg	cactggcctcacttcattcc	#37
*XBP1_unspliced*	ccgcagcactcagactacg	atgttctggaggggtgacaa	#62
*XBP1_spliced*	agttaagacagcgcttgggg	tgcacctgctgcggactcag	#37

First, expression intensities were calculated for each gene probed on the array for all hybridizations using Illumina's Beadstudio #1 software. Second, intensity values were quality controlled and normalized: quality control was carried out by using the Illumina Beadstudio detection P-value set to <b0.1 as a cutoff. This removed genes which were effectively absent from the array (i.e., were not detected). All the arrays were then quantile normalized using the normalize quantiles routine from the Affymetrics package in Bioconductor. This procedure accounted for any variation in hybridization intensity between the individual arrays. An assessment of several different normalization techniques using the Bioconductor maCorrPlot routine suggested that normalize.quantiles was the most appropriate for the data. Finally, these normalized data were imported into GeneSpring and analyzed for differentially expressed genes. The groups of biological replicates were described to the software, and significantly differentially expressed genes were determined based on t-tests and fold difference changes in expression level.

Hybridization and scanning of the second array followed the standard Illumina protocols. Illumina HT-12 Expression Bead Chips were normalized together using the quantile normalization algorithm without background subtraction. Probe sets with known bad quality and without EntrezID annotation were removed, thus resulting in 20811 EntrezID annotated genes. If multiple probes matched the same ID, those having the largest inter-quartile range were retained. Differential gene expression analysis between treatment groups was performed by using moderated t-statistics. The microarray data have been deposited at Gene Expression Omnibus (GEO) under the accession number GSE79630.

### Western blot

Cell cultures grown in 6-well plates were rinsed twice with ice-cold PBS and harvested by scraping in 500 μl PBS. After centrifugation (5 min, 200 g, 4°C) the cell pellet was homogenized in triple detergent buffer (50 mM Tris-HCl (pH 8), 150 mM NaCl, 0.02% (w/v) NaN_3_, 0.5% (w/v) sodium deoxycholate, 0.1% (w/v) SDS, 1% (v/v) Nonidet P-40) supplemented with protease inhibitors (1x complete protease inhibitor cocktail (Roche) and 1 mM PMSF). The homogenate was mixed on a vortex mixer and incubated on ice for at least 30 min or frozen. After a further centrifugation step (15 min, 4°C, 16100 g), the protein concentration of the protein-containing supernatant was estimated by the Bradford assay (Bio-Rad). Equal amounts (at least 17 μg) of total protein were loaded onto a 10% polyacrylamide gel and transferred to a nitrocellulose membrane. The following primary antibodies were used: anti-ERN1/IRE1alpha antibody (1:1000, Cell Signaling Technology, CST#3294), anti-ALPK1 antibody (1:300, Novus Biologicals, NBP1-83594), anti-β-actin antibody (1:5000; Sigma, #A5441). Relative protein expression was quantified using *Image J* software.

For β-casein detection, cells were washed in phosphate-buffered saline (PBS) and lysed in lysis buffer (50 mM Tris-HCl pH 8, 150 mM NaCl, 0.02% (w/v) NaN_3._ 0.5% (w/v) NaDeoxycholate, 0.1% SDS, 1% (v/v) NP40). About 50 mg of protein were separated by 10% sodium dodecyl sulfate polyacrylamide gel electrophoresis (SDS-PAGE) and transferred to nitrocellulose membranes for 2 hrs using the Tetra Cell-Blot (Biorad) with 1x blotting buffer (20 mM Tris, 150 mM glycine, 20% methanol, pH 8.3).

Western blot for β-casein detection was performed with mouse anti-β-casein antibody (Thermo/Pierce, F20.14, MAI-46056, 1:200) in 5% dry milk/TBS/Tween 20, followed by mouse anti-β-actin (Sigma, A5441; 1:5,000) species-specific secondary HRP-coupled antibody incubation (Jackson labs, 1:20,000). Protein bands were visualized using Amersham ECL Prime Western Blotting Detection Reagent (GE Healthcare) Chemidoc (BioRad) Quantification of western blots was done with ImageLab software (BioRad) or ImageJ by normalizing specific bands to β-actin.

### Colony formation

In 2D, cells analyzed for colony forming capacity were seeded in suitable dilutions (200-1000 cells) in technical triplicates on 12-well plates. After 5 days, cells were fixed in ice-cold methanol at −20°C for 15 min, stained with a 0.05% crystal violet solution for 3 min and rinsed 3 times with tap water. Cells were allowed to air-dry and counts were performed using a stereomicroscope. Only colonies with ≥6 cells were counted.

In 3D, 100 and 500 cells were embedded in a 1:1 Matrigel-Media (DMEM/10% FBS) mix using 96-well plates and the Matrigel was allowed to solidify. After 7 days colonies were counted under a stereomicroscope. For all depicted experiments at least three independent replicates were performed.

### Cancer stem cell spheroid assay (anchorage-independent assay in methyl cellulose)

Cells were dissociated to single cells by incubation in 0.05% trypsin-EDTA solution (Invitrogen, 25300-054) and re-suspended in serum-free medium (SFM) [DMEM-F12 (Invitrogen, 31331), 20 ng/ml EGF (R&D Systems, 236EG200), 0.4% bovine serum albumin (BSA, Sigma, A8412)], B27 Supplement (1:50, Invitrogen, 17504-044) and 4 mg/ml insulin (Invitrogen, 12585-014). For quantification of the sphere forming capacity, between 1000 and 9000 cells were seeded in SFM containing 1% methylcellulose (Sigma, M0512) into individual wells of poly(2-hydroxyethylmetacrylate) (Sigma, P3932) coated 96-well plates. After 7 to 10 days all spheres containing 4 or more cells were counted.

### Apoptosis assay

For detection of apoptosis, cells were stained with FITC Annexin V Apoptosis Detection Kit I (BD Bioscience) following the manufacturer's instructions. In brief, cells were collected with 0.05% trypsin–EDTA solution, washed, and diluted to 1 million cells per ml in 1x Binding Buffer. Annexin V staining was performed for 15 min at room temperature in the dark by adding 5μl FITC-coupled antibody solution and 5 μl propidium iodide (PI) to the cells in 100 μl 1x Binding Buffer. Afterwards 400 μl 1x binding buffer was added and cells were analyzed using a BD LSR Fortessa and BD FACS Diva Software (Becton Dickinson). A total of 10,000 cells were counted. Dot plots and histograms were generated with FlowJo software.

### Cell line specificity test

For the determination of cell line specificity of ERN1 or ALPK1 knockdown effects, 3 other breast cancer cell lines than MDA-MB-468 (MDA-MB-453, SKBR3 and BT474) were used. Three days after transfection with siRNA as described above, knockdown efficiency was confirmed via qPCR. If a sufficient knockdown was achieved, 200 cells per 6-well were seeded in triplicates for assessment of colony forming units and 100 cells per 96-well were seeded for the proliferation assay as described above.

### Statistics

The GraphPad Prism 6.0 statistical analysis program and Microsoft Excel 14.0 were used throughout. The raw data were processed to calculate the StDEV or SEM (as indicated by error bars in the figures). Significance is indicated by *, p<0.05; **, p<0.01; ***, p<0.001. Statistical significances of Figures 2A, 2B, 2E, 2F, 2G, 3A, 3B, 5D, S5C and S8 were evaluated by the two-tailed Student's *t-*test. The ordinary one-way ANOVA test was used for statistics in Figure 1D. For statistical significances of Figures 5A, 5B, 7A and 7B Tukey's test was used.

## SUPPLEMENTARY FIGURES AND TABLE


