# Supplementary material for: ERN1 and ALPK1 inhibit differentiation of bi-potential tumor-initiating cells in human breast cancer
Source: Oncotarget. 2016 Nov 4;7(50):83278–93. doi: 10.18632/oncotarget.13086 (PMC5347769; doi:10.18632/oncotarget.13086)
Supplement: Supplementary file 1 [file oncotarget-07-83278-s001.pdf]

ERN1 and ALPK1 inhibit differentiation of bi-potential tumor-initiating cells in human breast cancer

SUPPLEMENTARY FIGURES AND TABLE

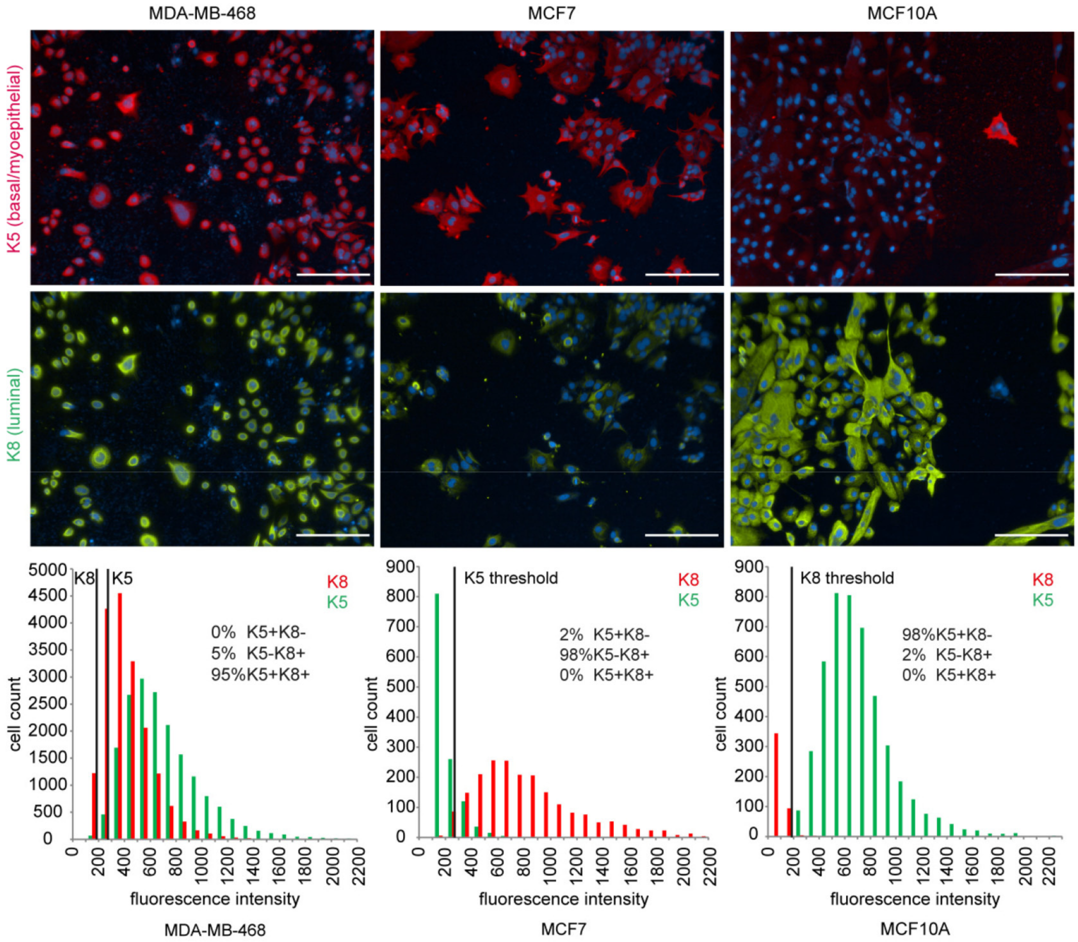

Supplementary Figure S1: Identification of fluorescent gate thresholds using defined luminal and myoepithelial cell lines MCF7 and MCF10A. Top: Immunofluorescence imaging for K5 (green) and K8 (red) on the indicated cell lines: MDA-MB-468, MCF7 and MCF10A. MCF7 harbors approx. 2% $K5^+$ cells and MCF10A on average 2% $K8^+$ cells. Scale bars represent 100 µm. **Bottom:** Quantification of fluorescence intensity by cell number confirms the identity and therefore the gate threshold for screening cells for luminal/myoepithelial differentiation (see threshold indicators, black lines).

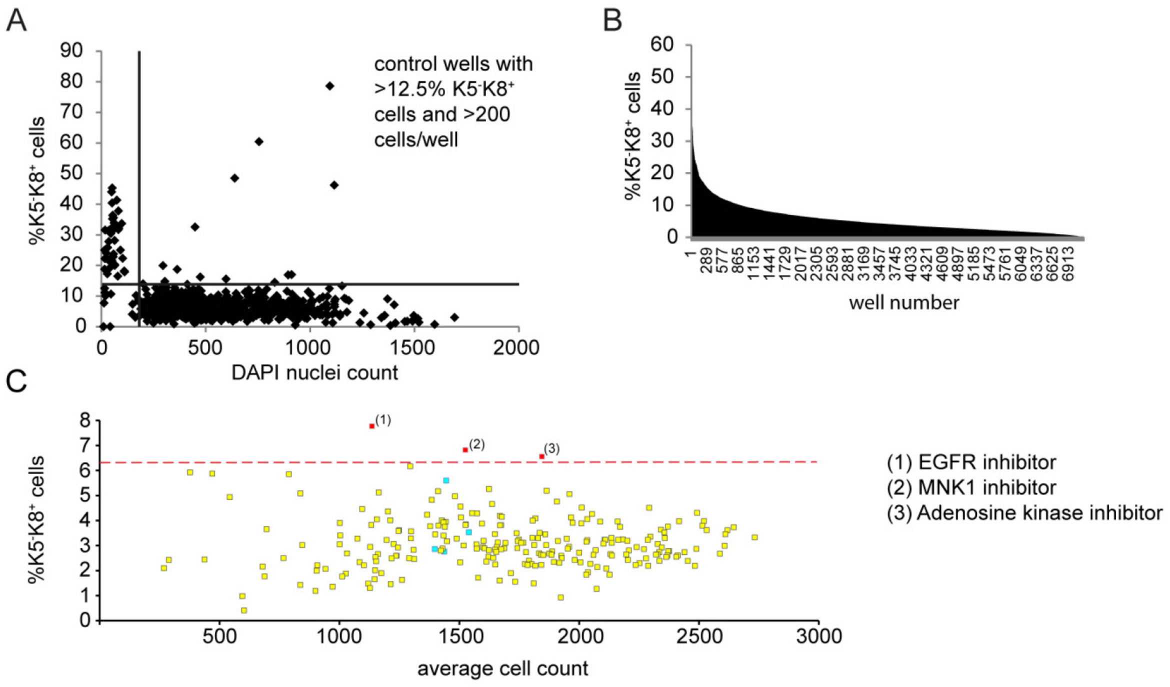

Supplementary Figure S2: Control data determine threshold for hit identification. A. %K8 single positive MDA-MB-468 cells in the non-specific control. These data were used to identify thresholds. Indicated are the threshold for dead/dying cells (vertical line) and the threshold for %K8 single positive cells identifying hits (horizontal line). **B.** %K8 single positive MDA-MB-468 cells over all treatments. **C.** Summary depiction of a kinase inhibitor screen on MDA-MB-468 cells. Individual dots represent averages of triplicate wells. Yellow indicates treatments that do not reach the threshold (2 standard deviations above the mean of all controls). Blue squares represent the controls. The red color indicates treatments clearing the threshold (EGFR=Epidermal Growth Factor Receptor, MNK1=MAP kinase interacting kinase 1).

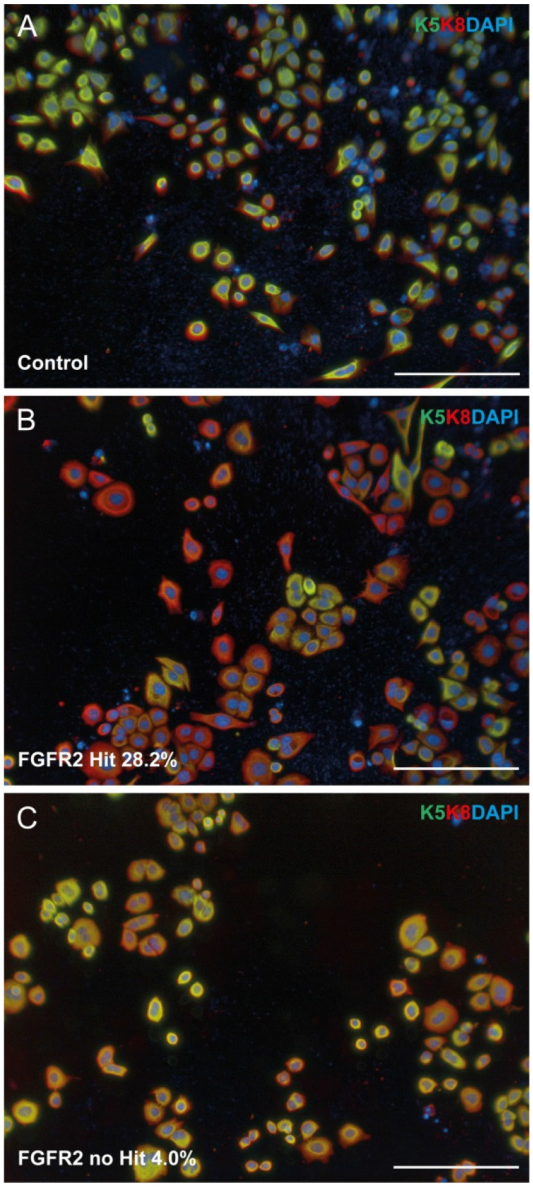

Supplemental Figure S3: FGFR2 is a double-hit in the screen. A-C. Immunocytochemical analysis of FGFR2 knockdown with individual shRNAs. MDA-MB-468 cells are marked with keratin 5 (K5, green), keratin 8 (K8, red) and DAPI (nuclei, blue). Depicted are cells transfected with a non-specific virus control (A) as well as a sample hit with a shFGFR2 virus (B) and a shFGFR2 non-hit (C). Scale bars represent 100 μm.

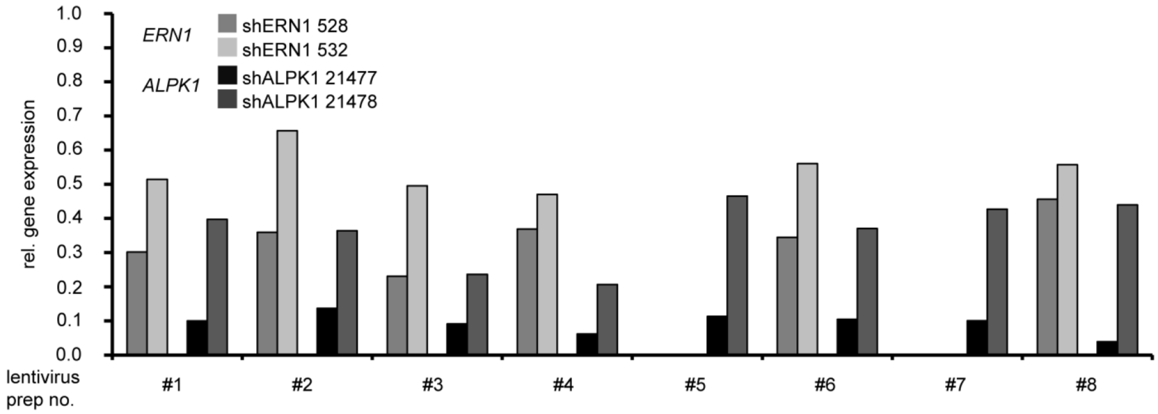

Supplementary Figure S4: qPCR data from lentiviral knockdowns. qPCR data of *ERN1* and *ALPK1* mRNA expression in MDA-MB-468 cells transduced with lentiviruses carrying vectors with the indicated shRNAs relative to empty vector (control). Expression values were normalized to *HPRT1*. *ERN1* and *ALPK1* expression in control cells was set to 1.

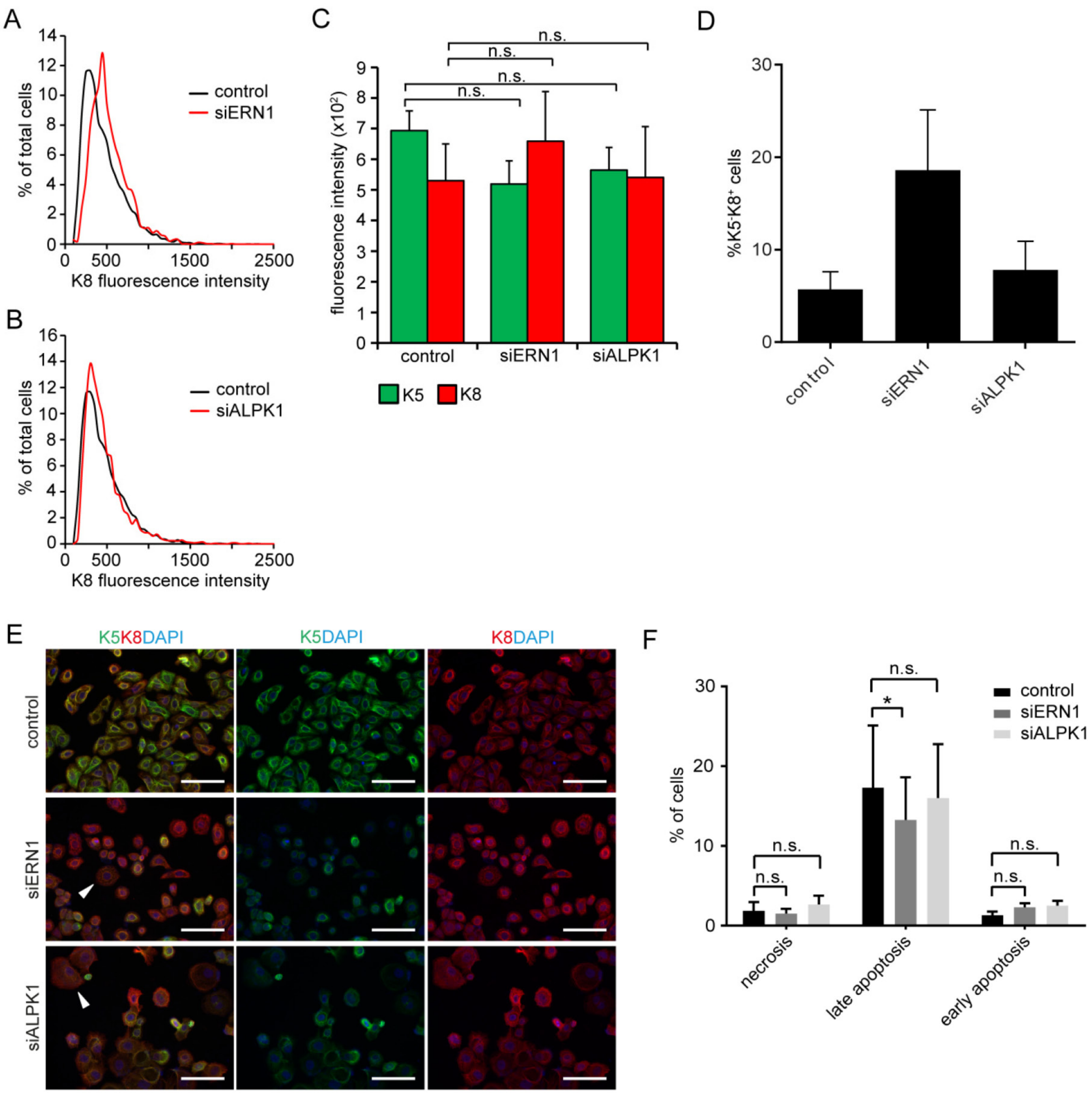

Supplementary Figure S5: Keratin patterns upon ALPK1/ERN1 knockdown in MDA-MB-468 cells. A, B. Profile of K8 cellular fluorescence intensities after ERN1 (A) and ALPK1 (B) knockdown compared to control cells. Depicted are representative curves. **C.** K5 and K8 expression in small (<830 μm²) cells after ERN1 or ALPK1 knockdown compared to controls (n=3). Values are the mean ± SEM. Statistical significances were evaluated by two-tailed Student's *t*-test. **D.** Percent K8 single positive MDA-MB-468 cells in control, siERN1 and siALPK1 treatment. **E.** Immunofluorescence staining of K5 (green) and K8 (red) in control cells (top row), ERN1 knockdown cells (middle row) and ALPK1 knockdown cells (bottom row). Nuclei are counterstained with DAPI (blue). Arrowheads indicate exemplary cells with increased size. Scale bars represent 100 μm. **F.** Quantification of Annexin V-PI flow cytometric analysis in MDA-MB-468 siERN1 and siALPK1 knock-down cells compared to siRNA transfected control cells, 3 days post-transfection (n=3).

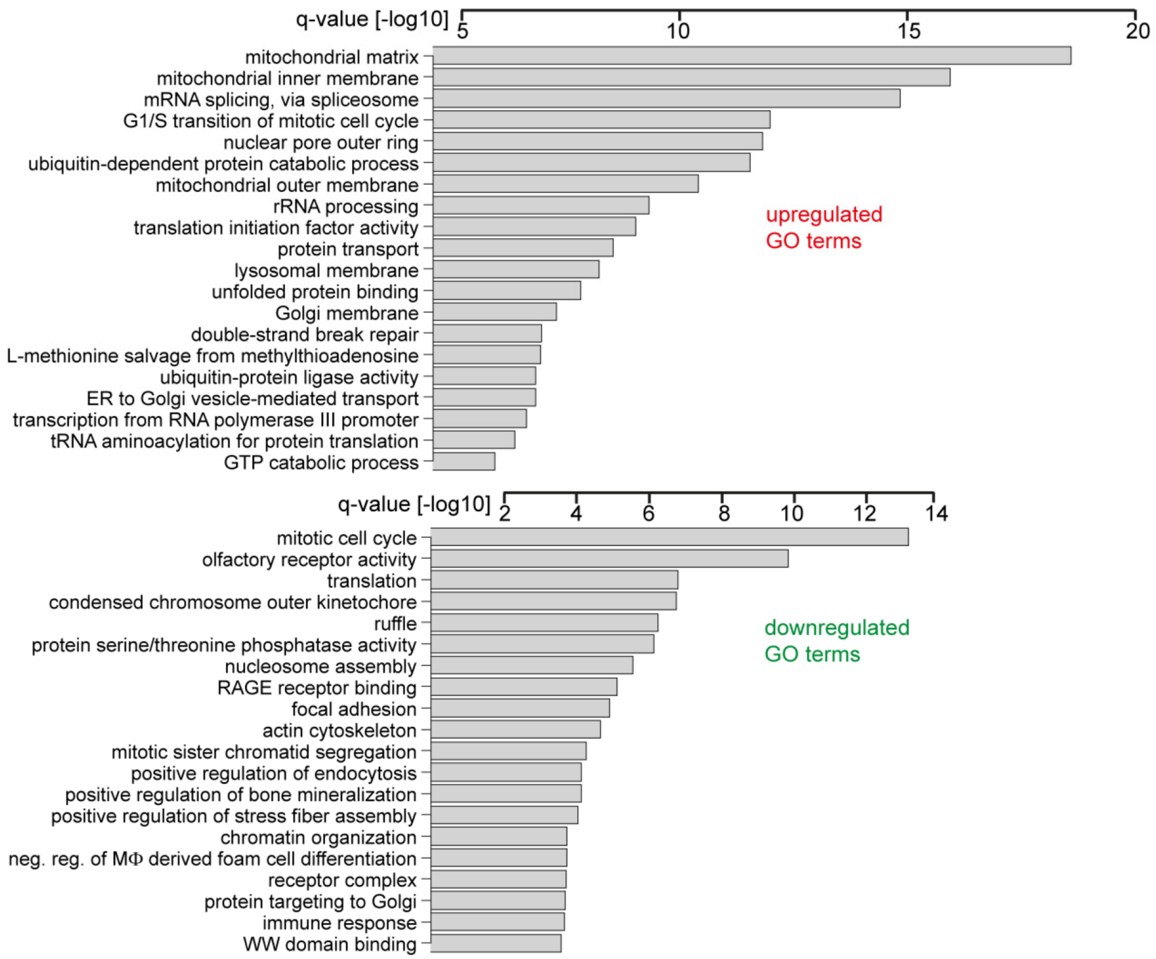

Supplementary Figure S6: Expression array analysis reveals differentiation pattern in ERN1 or ALPK1 knockdown MDA-MB-468 cells. A ranked-based gene set enrichment analysis of a siRNA microarray comparing ALPK1 knockdown cells to the respective controls (FDR corrected q-value <0.05). Biological processes from the human Gene Ontology were used as gene sets.

	log2 FC	gene name
myoepithelial	-0,13	*vimentin*
	-1,26	*tumor protein p63*
	-0,25	*alpha smooth muscle actin*
	-1,51	*keratin 5*
	-0,23	*keratin 6A*
	-0,72	*keratin 6B*
	-0,22	*keratin 6C*
	0,10	*keratin 14*
luminal	0,60	*keratin 8*
	0,73	*keratin 17*
	1,43	*keratin 18*
	0,38	*GATA binding protein 3*
	1,04	*mucin 1*
	0,23	*progesterone receptor*
	0,64	*prolactin receptor*

Supplementary Figure S7: Differentiation marker regulation upon siALPK1 treatment of MDA-MB-468 cells. Log fold change in marker genes for the myoepithelial and luminal lineage after siRNA-mediated knockdown of ALPK1 extracted from microarray data.

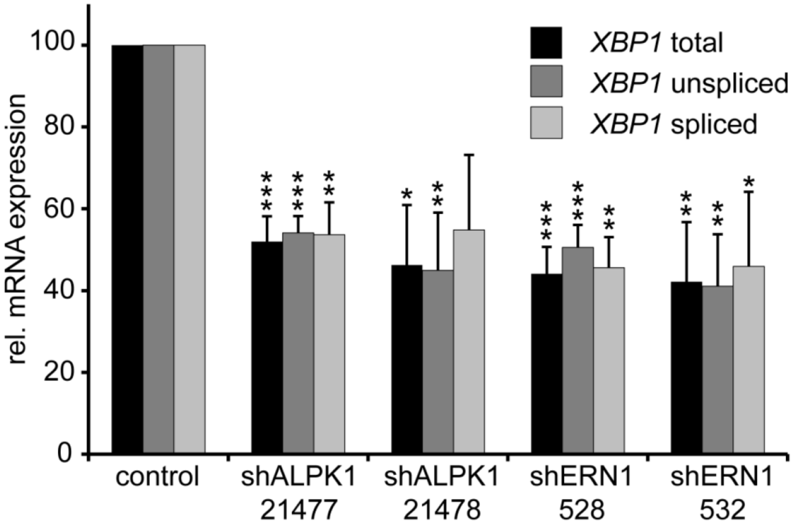

Supplementary Figure S8: *XBP1* levels upon ALPK1 or ERN1 knockdown in MDA-MB-468 cells. qPCR data of *XBP1* total, *XBP1* spliced and *XBP1* unspliced RNA upon knockdown of ALPK1 or ERN1 using shRNA ($n \geq 3$). Expression values were normalized to *HPRT1*. *XBP1* total and isoform expression in control cells was set to 1. Values are the mean ± SEM. Statistical significances in this figure were evaluated by two-tailed Student's *t*-test.

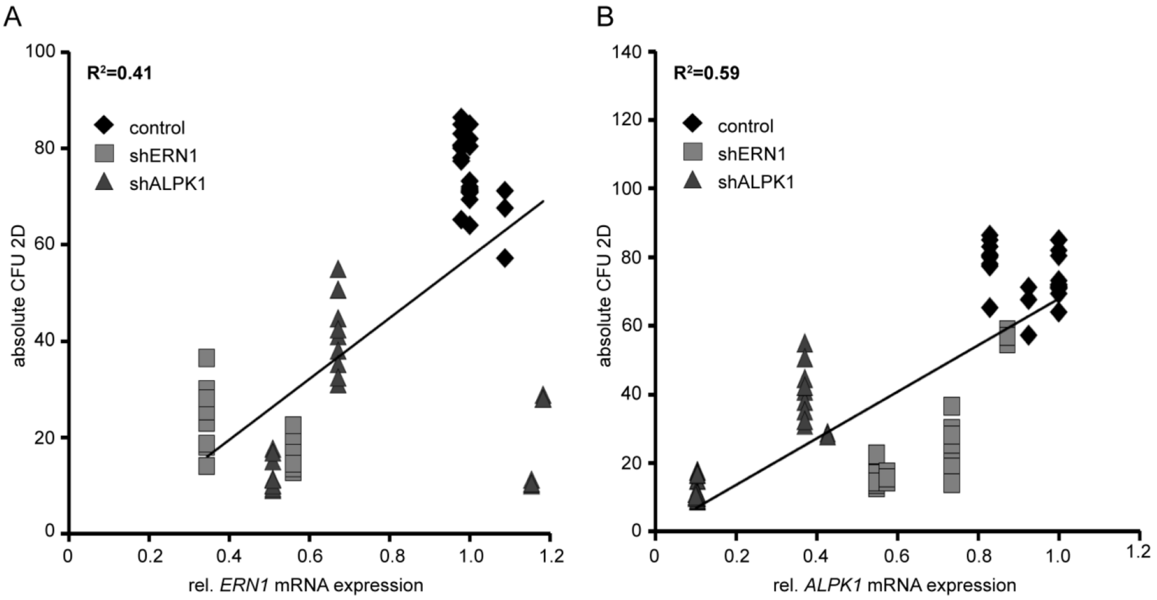

Supplementary Figure S9: Correlation of colony forming ability and knockdown efficiency by shRNA for *ERN1* and *ALPK1*. A, B. Regression graph describing the correlation of relative *ERN1* or *ALPK1* mRNA expression in MDA-MB-468 cells with observed colony forming units (CFU) in 2D. R^2 describes the coefficient of determination.

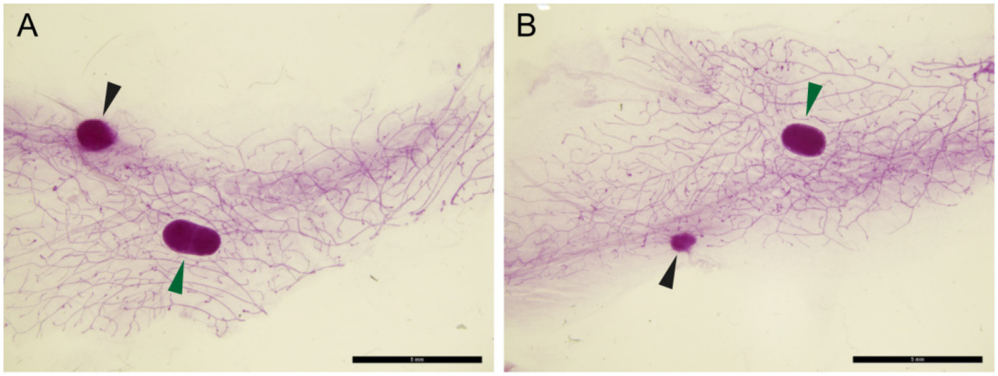

Supplementary Figure S10: Exemplary tumor growths in the mammary gland of transplanted animals of ERN1 or ALPK1 knockdown MDA-MB-468 cells. A, B. Exemplary tumor growths in the mammary gland of transplanted animals of ERN1 (A) and ALPK1 (B) knockdown cells. Black arrowheads indicate the tumor growth within the mammary gland. Green arrowheads indicate the lymph node. Scale bars represent 5 mm.

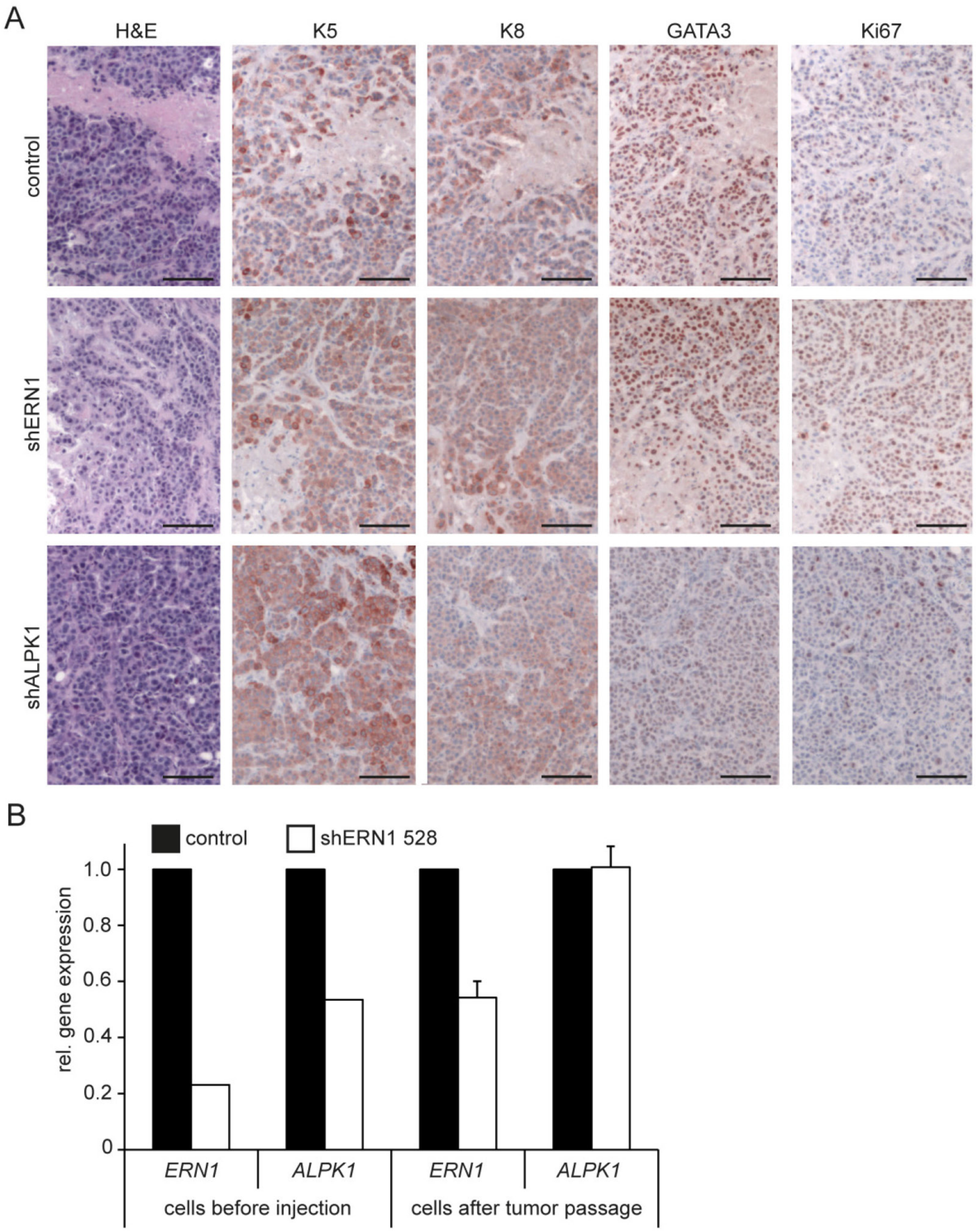

Supplementary Figure S11: Late ERN1 and ALPK1 knockdown tumors do not differ in expression pattern or phenotype from control tumors. A. The panel depicts exemplary images of tumors derived from control, shERN1 and shALPK1 treated MDA-MB-468 cells (from top to bottom). Histochemical stainings are indicated on the top from left to right: H&E (hematoxylin and eosin), K5 (keratin 5), K8 (keratin 8), GATA3 and Ki67. Scale bars represent 100 µm. **B.** qPCR analysis of the targeted kinases in the cells before injection and after tumor formation. The bars depicting the kinase expression after tumor passage represent a combination (n=2) of values from 5×10^5 cell transplants. Values are the mean ± SEM. Statistical significances in this figure were evaluated by Tukey's test.

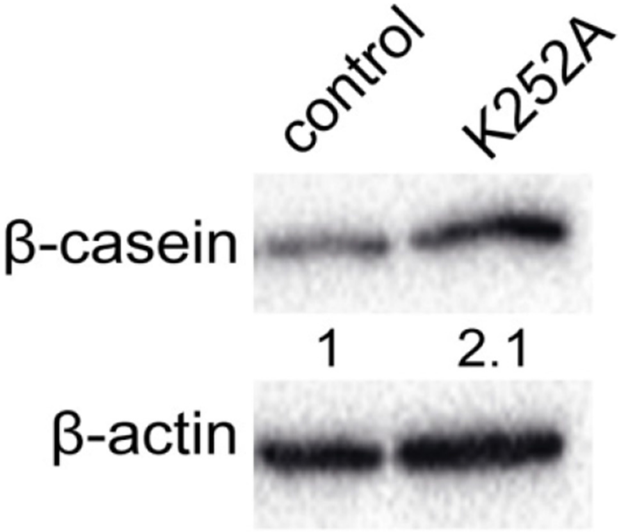

Supplementary Figure S12: K252a induces β-casein production. Representative Western blots detecting β-casein in MDA-MB-468 cells transfected with siRNA targeting ERN1 and ALPK1, respectively. β-actin was used as loading control.

Supplementary Table S1: Average percentage of Keratin 8 positive (Keratin 5 negative) cells under treatment with individual kinase knockdown

siRNA ID	GeneSymbol	avg %K8	STD %K8
TRCN0000021478	ALPK1	16,372	0,753
TRCN0000021477	ALPK1	14,992	3,675
TRCN0000055425	BTK	19,545	4,188
TRCN0000000359	BTK	12,691	2,756
TRCN0000000688	CDK10	21,289	3,520
TRCN0000000689	CDK10	16,912	3,343
TRCN0000006242	CDK17	18,412	3,044
TRCN0000006245	CDK17	18,160	18,289
TRCN0000000532	ERN1	15,329	6,214
TRCN0000000528	ERN1	15,240	3,377
TRCN0000000370	FGFR2	28,244	5,243
TRCN0000000367	FGFR2	19,638	3,280
TRCN0000000548	IRAK2	15,611	3,148
TRCN0000000551	IRAK2	13,025	1,155
TRCN0000006265	PDK4	18,882	7,735
TRCN0000006267	PDK4	12,916	5,255
TRCN0000037507	RIOK2	16,084	6,577
TRCN0000037508	RIOK2	14,613	2,390
TRCN0000002274	STK32B	14,823	6,339
TRCN0000002276	STK32B	13,304	3,361
TRCN0000121179	TXK	17,812	2,351
TRCN0000001578	TXK	12,821	2,046
	controls	4,122	3,016

Shown are (from left to right) identifiers for the used siRNA vectors, the targeted kinase as well as the effect on Keratin 8 positive cells with standard deviation.
